# A Stand-Alone Wearable Measurement System for Continuous Hand Tremor Monitoring

**DOI:** 10.3390/s26185949

**Published:** 2026-09-20

**Authors:** Laura Arruzzoli, Carmen Galletta, Giovanni Gugliandolo, Mariangela Latino, Giovanni Crupi, Cristiano De Marchis, Nicola Donato

**Affiliations:** 1 Department of Biomedical and Dental Sciences and Morphofunctional Imaging, University of Messina, 98125 Messina, Italy; crupig@unime.it; 2 Department of Engineering, University of Messina, 98166 Messina, Italy; carmen.galletta1@studenti.unime.it (C.G.); giovanni.gugliandolo@unime.it (G.G.); cristiano.demarchis@unime.it (C.D.M.); nicola.donato@unime.it (N.D.); 3 Institute for Chemical-Physical Processes, National Research Council of Italy (IPCF-CNR), Viale Ferdinando Stagno d’Alcontres, 98158 Messina, Italy; mariangela.latino@cnr.it

**Keywords:** biomedical monitoring, inertial sensors, neurodegenerative diseases, spectral estimation, tremor analysis, wearable measurement systems

## Abstract

Progressive neurodegenerative diseases are often associated with motor symptoms, among which tremor is one of the most debilitating manifestations, significantly compromising daily activities and quality of life. Continuous, non-invasive monitoring of tremor characteristics helps to assess disease progression and optimize treatment strategies. For this reason, wearable measurement systems can provide an objective and non-invasive means for the quantitative characterization and monitoring of tremor-related movements. This study addresses the development of a compact, low-power wearable board for real-time tremor monitoring based on the Nicla Sense ME embedded system. The proposed device performs on-board processing to extract spectral features of movement and wirelessly sends them via Bluetooth Low Energy to a remote host, enabling on-demand visualization or storage of tremor data. The measurement system was technically validated through a comparative experimental protocol based on the simultaneous acquisition of tremor signals using the proposed device and a commercial Shimmer3 IMU as the reference system. The comparison considered both the similarity of the measured spectrograms and the agreement in dominant-frequency estimation. Spectrogram correlation coefficients reached 0.85, with amplitude MAE and RMSE values as low as 0.07 m/s^2^ and 0.14 m/s^2^, respectively. For dominant-frequency estimation, Pearson correlation coefficients reached 0.99, with MAE and RMSE values as low as 0.17 Hz and 0.20 Hz. These results support the feasibility of the proposed architecture as a practical wearable solution for quantitative hand tremor monitoring.

## 1. Introduction

Neurodegenerative diseases represent one of the most complex challenges for modern healthcare systems, mainly due to the progressive aging of the global population. Among them, Parkinson’s disease (PD) stands out as the second most widespread neurodegenerative disorder, after the Alzheimer’s disease (AD), with a rapidly increasing prevalence [1]. The disease is characterised by a wide range of symptoms, but the main clinical markers for diagnosis and therapeutic management are motor symptoms, particularly bradykinesia, rigidity and tremor [2]. The traditional clinical assessment of tremor is still largely based on subjective scales, such as the MDS—Unified Parkinson’s Disease Rating Scale (MDS-UPDRS) [3], which are affected by inter- and intra-observer variability and do not allow continuous monitoring of symptom progression. As a result, this makes it impossible to capture the fluctuations in complex motor data and the “on–off” phenomena that occur in the daily lives of individuals with PD. In recent years, wearable measurement systems have seen considerable expansion, opening new opportunities for cost-effective telemedicine. This approach not only allows for constant and non-invasive monitoring of physiological parameters in household environments [4,5,6] but also contributes to a significant reduction in healthcare costs by minimizing the need for frequent in-person clinical visits. In this context, the use of inertial sensors integrated into portable devices, in particular micro-electro-mechanical systems (MEMSs) accelerometers and gyroscopes, has enabled the quantitative characterization of motor symptoms, allowing for the evaluation of disease progression and the effectiveness of pharmacological therapies [7,8,9,10].

In the literature, various approaches have been proposed for acquiring tremor data, which can be categorized based on the processing architecture and type of device. These approaches highlight how frequency analysis of accelerometer signals, using Fourier transforms or time–frequency techniques, allows the characteristic bands of pathological tremor to be identified and distinguished from those of physiological tremor or other components of voluntary movement [11]. A first line of research exploits the use of smart devices for the clinical assessment of symptoms [12,13,14,15]. In Ref. [12], the three-axis accelerometer and three-axis gyroscope integrated into the Apple Watch Series 3 were used to acquire data from 21 patients with PD and 27 healthy subjects, demonstrating how these devices can be used in a complementary way for monitoring and evaluating the disease. A second approach involves the use of dedicated custom wireless platforms, which allow direct access to raw data and greater flexibility in configuring sampling parameters [12,16,17,18]. In Ref. [19], a system based on the LinkIt ONE platform was proposed, equipped with multiple connectivity and global positioning systems (GPSs), to which four accelerometers were connected via an interface board. This configuration allows for a more detailed analysis of tremors and has demonstrated the effectiveness of long-term remote monitoring on 41 idiopathic parkinsonian subjects. Similarly, in [18], a wearable inertial measurement system based on triaxial accelerometers was adopted to monitor tremor in 12 patients with Parkinson’s disease. Data pre-processing was performed by extracting information through the tremor spectrum, highlighting the relevance of spectral characteristics for the objective assessment of Parkinson’s tremor in real conditions. Data were then analyzed using classification algorithms.

Nevertheless, these systems are limited to recording and transmitting raw data to cloud platforms for processing. Continuous transmission of raw inertial data requires a persistent wireless connection and may increase communication-related energy consumption and the amount of data transferred to the remote processing unit. To overcome these barriers, research is shifting toward the Edge Computing paradigm, which involves moving algorithmic processing directly to the sensor node [20,21]. The integration of low-power microcontrollers with on-board signal processing capabilities allows relevant information to be processed locally, reducing the need for continuous raw data transmission and enabling the wireless interface to be activated only when data transfer is required.

In this context, the present study proposes the development of a compact wearable measurement system for tremor monitoring, based on the Nicla Sense ME platform, together with its metrological validation through a comparison with a commercial reference device. The comparison was aimed at assessing the agreement between the two systems in the estimation of the main tremor parameters. Unlike previous systems described in the literature, the proposed device exploits the computational capability of the integrated processor to perform on-edge frequency analysis using Fast Fourier transform (FFT), storing the processed spectral information and transmitting it via Bluetooth Low Energy (BLE) on demand. This approach allows the extraction of meaningful tremor descriptors directly on the device, enabling robust identification of the dominant frequency component of the tremor, while reducing raw data transmission and preserving clinically relevant parameters. The main objective of this work is to validate the metrological performance of the proposed system through a direct comparison with a commercial reference instrument, the Shimmer3 IMU, which is widely used in clinical movement research [22,23,24]. Through an experimental protocol that simulates tremors at physiological and pathological frequencies (2–7 Hz), it will be demonstrated that the developed system provides performance comparable to high-end instrumentation, while maintaining low costs and a minimally invasive form factor, thus meeting current needs in personalized and remote medicine. It is worth noting that the Shimmer3 IMU was used as a practical reference instrument because of its widespread use in wearable motion-analysis applications. Therefore, the comparison presented in this work should be interpreted as a relative validation against an established commercial platform. Accordingly, the validation presented in this study should be regarded as a technical comparative assessment of the developed measurement system rather than as a clinical validation. Clinical validation represents an important subsequent step, but it is beyond the scope of the present contribution.

The novelty of the present work lies in combining a compact and low-cost wearable platform with on-board spectral processing, local storage, and on-demand BLE transmission, avoiding the need for continuous raw data streaming during acquisition. In addition, the present work specifically addresses the technical comparison of the developed system against an established commercial IMU, with particular attention to the estimation of tremor-related spectral parameters.

The remainder of the paper is organized as follows. Section 2 describes the hardware architecture based on the Nicla Sense ME, detailing the firmware implementation for edge-computing spectral analysis, and the experimental protocol adopted for the comparative validation. Section 3 presents the experimental results, including the spectrogram comparison and the dominant-frequency analysis performed against the reference device. Section 4 discusses the experimental findings and compares the proposed system with representative solutions reported in the literature. Finally, Section 5 summarizes the main conclusions, current limitations, and future developments.

## 2. Materials and Methods

### 2.1. System Architecture

The proposed wearable measurement system is implemented on the Arduino Nicla Sense ME development board (Qualcomm Incorporated, San Diego, CA, USA), which was selected for its compact form factor and suitability for low-power, stand-alone wearable sensing. The Nicla Sense ME integrates the Bosch Sensortec BHI260AP, a self-learning smart sensor featuring an embedded six-axis IMU, comprising a 16-bit tri-axial accelerometer and a 16-bit tri-axial gyroscope [25]. In this work, the embedded tri-axial accelerometer is used to capture hand tremor dynamics; its main technical specifications are reported in Table 1.

The Nicla board is based on the Nordic nRF52832 System On Chip (Nordic Semiconductor ASA, Trondheim, Norway), integrated into the ANNA-B112 stand-alone Bluetooth 5.0 Low Energy module (u-blox, Thalwil, canton of Zürich, Switzerland), which provides a 64 MHz Arm Cortex M4 microcontroller and Bluetooth 5 to support local processing and wireless data transfer. The device is powered by a rechargeable 3.7 V Li-Po battery (150 mAh) (MikroElektronika, Belgrade, Serbia), enabling stand-alone operation. The battery can be recharged through the battery management system integrated into the Nicla board; therefore, it can be recharged by simply connecting the board to a power source using a micro-USB cable.

**Figure 1:**
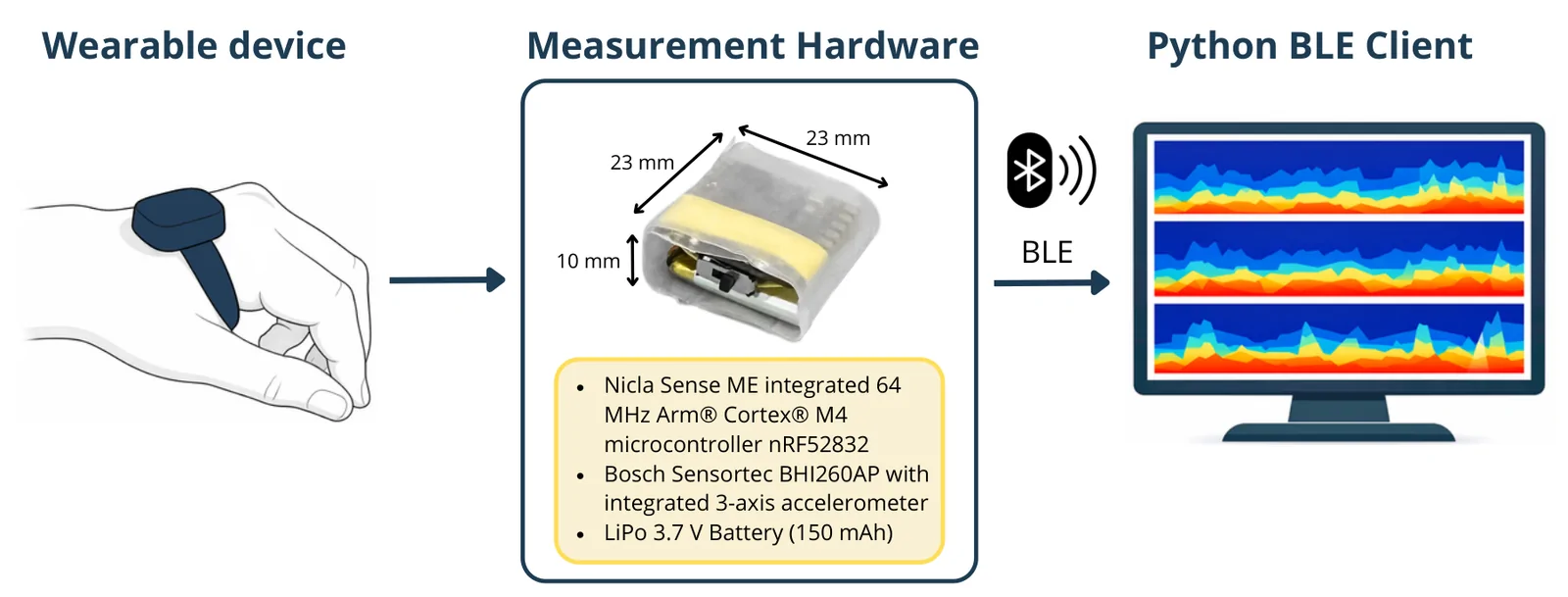
Block diagram of the proposed wearable tremor-monitoring system.

The prototype was assembled by soldering the battery leads to the Nicla board through a mechanical switch, which enables the device to be powered on and off. The battery and the switch were mechanically secured to the board using heat-shrink tubing. A photograph of the final prototype is shown in the inset of Figure 1. It is characterized by dimensions of approximately 23×23×10 mm^3^ and a mass of about 6.3 g, which is advantageous for comfortable use in wearable scenarios.

A block diagram of the proposed wearable tremor-monitoring system is shown in Figure 1. The system architecture follows a two-stage pipeline consisting of on-board acquisition and processing, and off-board data reception and visualization on a remote PC. On the wearable node, the firmware controls inertial-data acquisition, organizes samples into fixed-length windows, computes a frequency-domain representation for each window, and stores the resulting spectra in the on-board 2 MB flash memory. Specifically, for each window, the firmware acquires tri-axial acceleration samples (X, Y, Z). A Hamming window is applied to each time segment to reduce spectral leakage caused by the finite observation window, and the FFT is then computed independently for each axis. The complex FFT outputs are converted into amplitude spectra, producing one spectrum per axis and per time window. The computed spectra are appended to a file in flash memory managed through the LittleFS file system. This design enables continuous acquisition and local buffering of spectral data without requiring a persistent wireless link during recording.

Wireless communication is activated when the prototype establishes a BLE connection with a remote central device (e.g., a PC). Upon connection, the firmware switches from acquisition mode to transmission mode: spectra previously stored in flash memory are read back and transmitted over BLE using a custom service and characteristic with notifications enabled. To comply with the limited payload size of BLE packets, each spectrum is fragmented into small blocks before transmission. Once transmission is completed, the file containing the stored spectra is removed from flash memory, and the internal spectrum counter is reset, freeing storage resources for subsequent acquisitions.

On the PC side, a dedicated Python (v. 3.14.1) script acts as a BLE client to connect to the prototype, subscribe to characteristic notifications, and reconstruct the received spectra. The script accumulates incoming packets until a full spectrum is received, and then appends it to the corresponding axis-specific spectrogram buffer. At the end of the reception, the script saves the reconstructed spectral data to a CSV file and visualizes the three spectrograms (one per axis).

**Figure 2:**
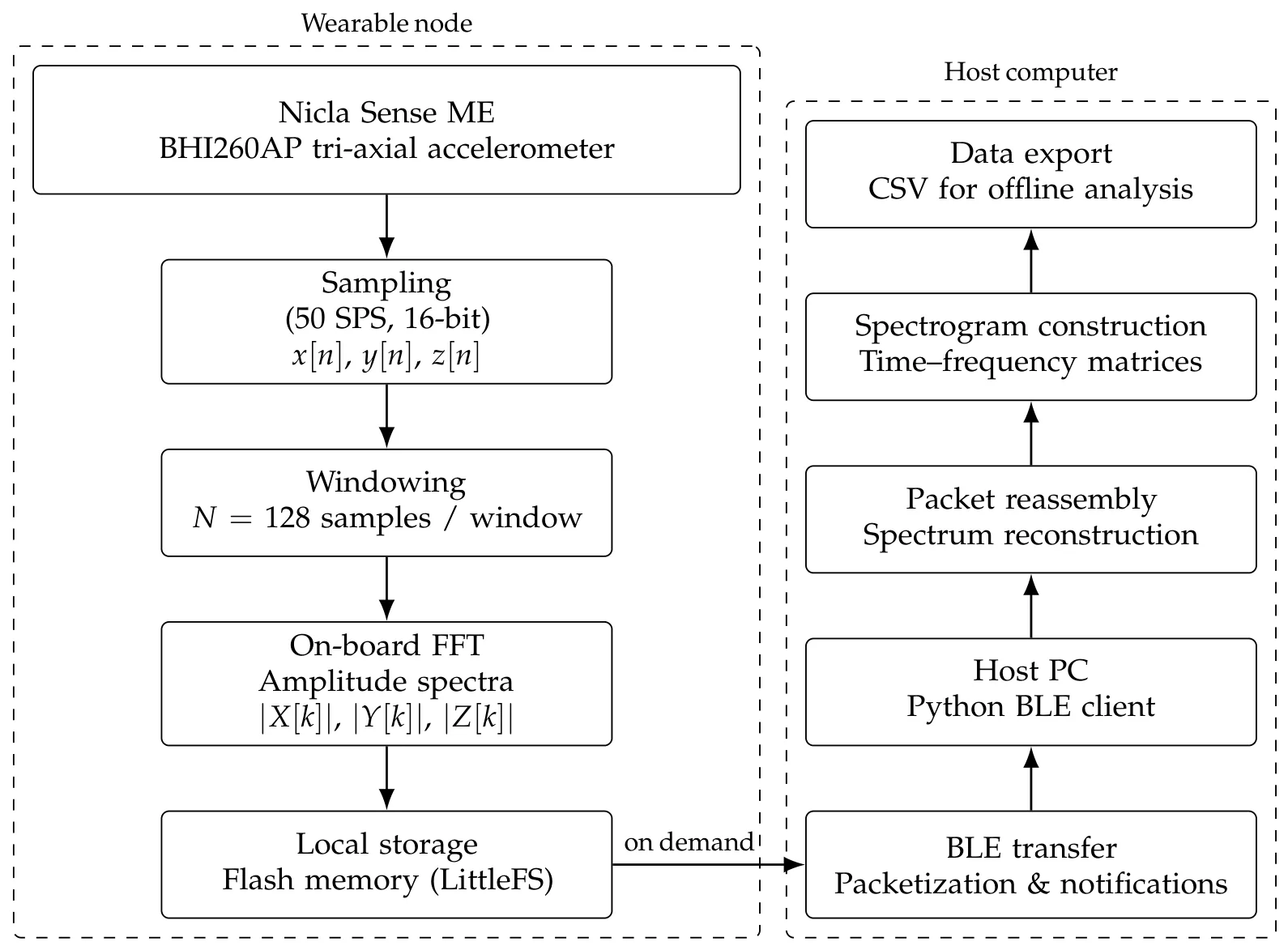
Block diagram of the data acquisition and processing pipeline. Tri-axial acceleration is sampled on the wearable node, processed on-board via windowing and FFT to obtain amplitude spectra, stored locally, and subsequently transferred via BLE to a host computer for spectrum reconstruction, spectrogram construction, and data export for offline analysis.

A detailed block diagram of the data acquisition and processing pipeline is reported in Figure 2.

### 2.2. Validation Protocol

The validation aimed to quantify the agreement between the proposed wearable prototype for tremor monitoring and a commercially available inertial sensing unit used as a reference, the Shimmer3 IMU (Shimmer Research, Dublin, Ireland) [26]. The comparison was performed to verify whether the prototype can reproduce, with adequate fidelity, the time–frequency content of hand oscillations and the corresponding dominant tremor frequency estimated from tri-axial accelerometric signals.

Table 2 summarizes the main specifications of the Shimmer3 IMU and the developed prototype. The prototype has substantially smaller dimensions and lower weight, which is advantageous for wearable applications in terms of user comfort. Although the current prototype version features a lower battery capacity, its estimated battery life is 43% longer than that of the Shimmer3 IMU, enabling operation for most of the day. The average current consumption of the prototype was experimentally measured to be approximately 7 mA during normal operation. Considering the nominal capacity of the employed 3.7-V, 150-mAh Li-Po battery, this corresponds to an estimated operating time of approximately 20 h.

It is worth noting that, in the current implementation, the main limitation for continuous operation without data transfer to a PC is the available memory capacity, which allows recording for up to approximately 5 h. The current implementation stores 32-bit spectral data for the three accelerometer axes. Considering the available 2 MB flash memory, this corresponds to approximately 5 h of continuous recording. However, this could be mitigated by implementing lightweight compression algorithms directly on the node [19].

Both devices integrate a 16-bit accelerometer with the same programmable full-scale ranges. Finally, compared to the reference device, the proposed system has a substantially lower cost. It should be noted that the Shimmer3 IMU is a more general-purpose platform, integrating additional sensors and offering a wider range of functionalities. Therefore, the comparison is intended to highlight the suitability of the proposed system for the specific application considered.

For the validation procedure, both measurement systems were configured with the same accelerometer range (±4 g) and sampling frequency (50 Hz). The sampling frequency was selected according to the frequency range targeted in this study. In particular, the experimental protocol focused on hand oscillations up to approximately 7 Hz, which includes the typical frequency range associated with Parkinsonian tremor. Therefore, a sampling frequency of 50 Hz provides an adequate margin for the frequency components of interest while limiting the computational burden associated with on-board processing and reducing flash-memory usage. A 50 Hz sampling rate is also commonly adopted in comparable wearable systems for tremor assessment [20,27].

For each analysis window, 128 samples were acquired per axis, processed via FFT, and converted to amplitude spectra. Each window, therefore, corresponds to 2.56 s of acceleration data. The 128-sample window length was selected as a compromise between frequency resolution and temporal resolution, providing a nominal frequency-bin spacing of approximately 0.39 Hz while preserving the ability to track temporal variations in the oscillation frequency. Moreover, the power-of-two window length enables an efficient FFT implementation on the embedded processor. A Hamming window was applied before FFT computation to reduce spectral leakage associated with the finite observation interval and with oscillations that are not necessarily periodic within each analysis window.

In the prototype, frequency-domain processing was performed on-board, as described in the Section 2.1, and the spectra were stored in the device flash memory. After acquisition, they were transferred to a PC via Bluetooth. Shimmer3 IMU data were streamed in real time via Bluetooth to the Shimmer ConsensysPRO software (v. 1.6.0), which was used to configure the acquisition and visualize the tri-axial signals. After the measurement sessions, the Shimmer3 IMU data were imported into MATLAB (v. R2026a) and processed using the same workflow adopted by the prototype device. The validation metrics were then extracted by comparing the results obtained from the two measurement systems.

Owing to the different data-transfer architectures of the two systems, the comparison was not based on a common hardware trigger. Temporal alignment was therefore performed offline before calculating the comparison metrics. The relative time offset between the two recordings was automatically estimated by reconstructing 128-sample spectral windows from the raw Shimmer3 signals and searching for the delay that minimized the mean cosine distance between the normalized three-axis spectral representations of the two systems within the investigated band. The search was performed with a temporal resolution of 20 ms, corresponding to one sample at the adopted 50 Hz sampling frequency.

**Figure 3:**
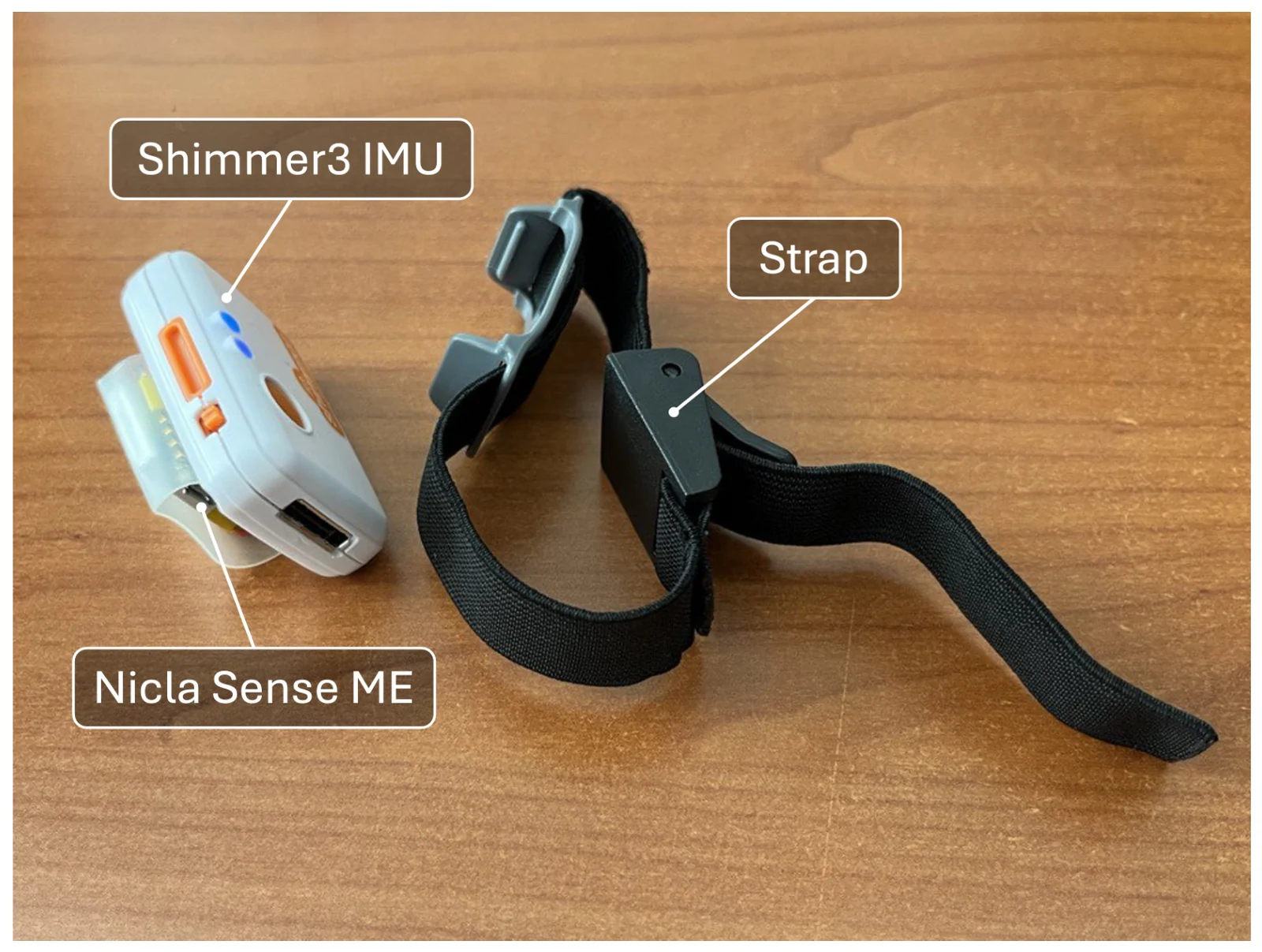
Photograph of the wearable prototype rigidly coupled to the Shimmer3 IMU and the strap used during the measurement sessions.

During the validation experiment, the prototype and the Shimmer3 IMU were rigidly fixed together so that both devices experienced the same motion and, consequently, similar acceleration variations over time (Figure 3). The coupled devices were worn on the dorsum of the right hand using a strap.

The experiment involved two healthy adult subjects, one 21-year-old female and one 39-year-old male (i.e., Subject 1 and Subject 2), both seated during acquisition. The study was conducted in accordance with the ethical principles of the Declaration of Helsinki. The experimental activity consisted exclusively of non-invasive inertial measurements of voluntary hand movements performed by healthy adult volunteers, who are also among the authors of the manuscript. Written informed consent was obtained from all participants before data acquisition.

Each subject participated in one measurement session lasting approximately 200 s. Each session consisted of five phases in which the hand oscillation frequency was progressively varied according to the following phases:

- Phase 1: light oscillation with frequency below 2 Hz;
- Phase 2: slow and controlled motion with frequency slightly higher than Phase 1;
- Phase 3: oscillations reaching approximately 5 Hz;
- Phase 4: simulated tremor reaching 7 Hz (within the typical 4–7.5 Hz range associated with Parkinsonian tremor);
- Phase 5: rapid reduction of oscillation frequency returning toward the initial low-frequency condition.

The voluntary hand oscillations adopted in this protocol were not intended to reproduce the full kinematic complexity of pathological tremor. The protocol was instead designed to expose the prototype and the reference system simultaneously to the same controlled hand motion over the frequency range of interest, thus enabling a technical comparison of their measurement outputs.

After the measurement sessions, a total of 52 paired measurement windows were analyzed for Subject 1 and 57 for Subject 2.

**Figure 4:**
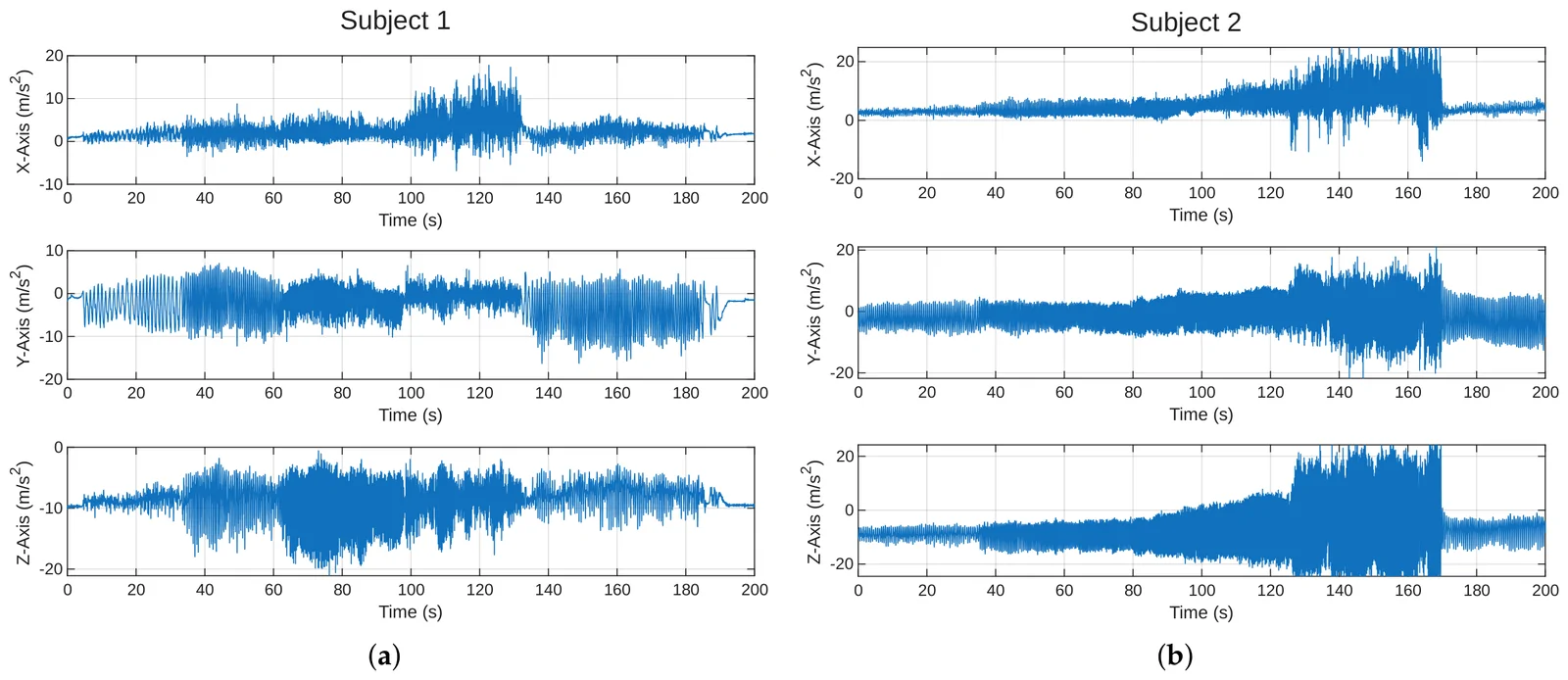
Time-domain tri-axial acceleration signals acquired during the validation protocol for two measurement sessions: (**a**) Subject 1 and (**b**) Subject 2. Measurements were acquired using Shimmer3 IMU.

Figure 4 shows the time-domain tri-axial acceleration signals acquired with the Shimmer3 IMU for Subject 1 and Subject 2. The recordings clearly reflect the five phases of the validation protocol, with distinct changes in oscillation amplitude and frequency across the successive phases.

A qualitative comparison was first performed by visually inspecting the spectrograms produced by the two systems, where time is reported on the horizontal axis, frequency is reported on the vertical axis, and spectral amplitude is encoded by a colormap. For the quantitative comparison of the spectrograms, the Pearson correlation coefficient (*r*), the mean absolute error (MAE), and the root mean square error (RMSE) were calculated for each subject and accelerometer axis.

Moreover, for each time window, the dominant oscillation frequency was estimated for both the prototype and the reference system. The agreement between the resulting dominant-frequency trajectories was quantified using the Pearson correlation coefficient, the Bland–Altman bias, the standard deviation of the error, MAE, RMSE, and the corresponding 95% limits of agreement. The results of these comparisons are reported in Section 3.

## 3. Results

**Figure 5:**
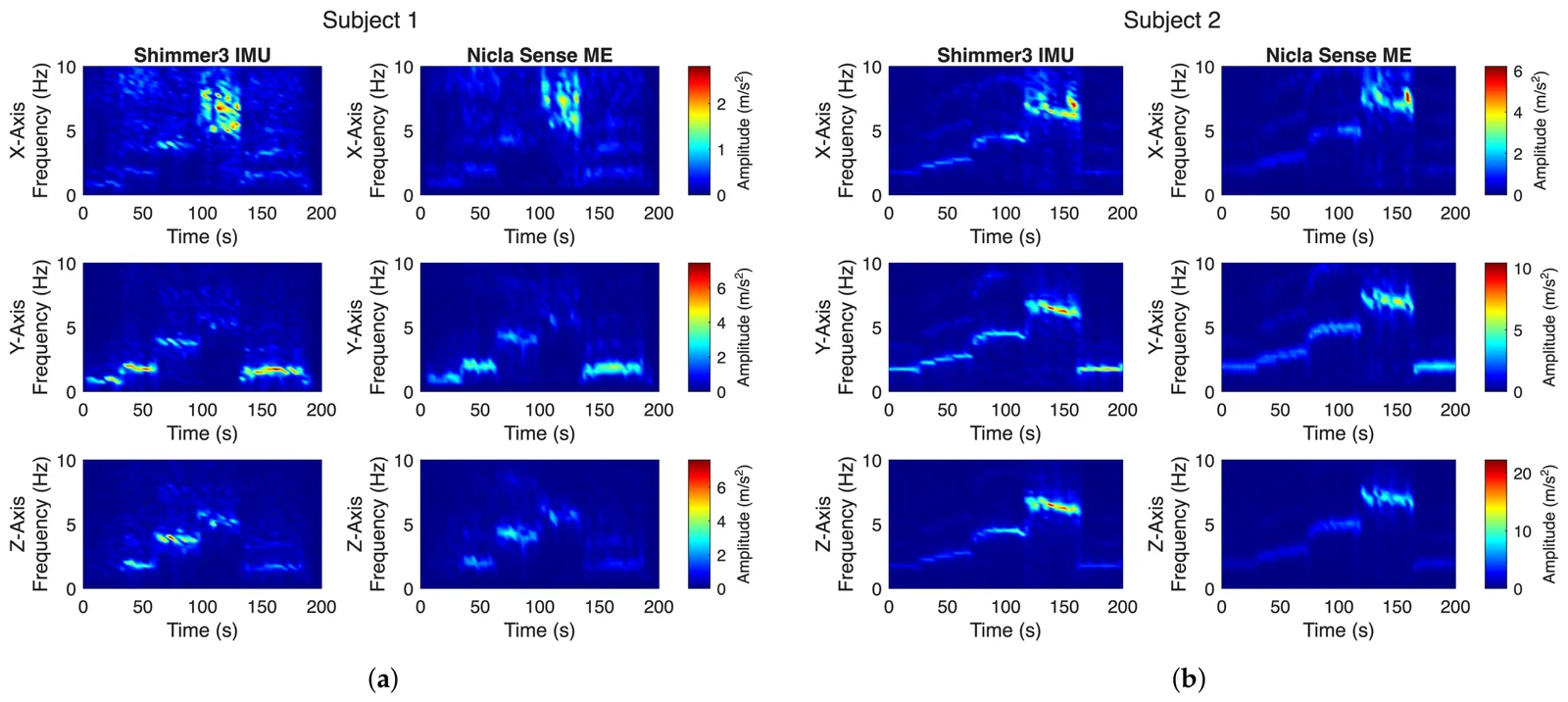
Time–frequency spectrogram comparison for (**a**) Subject 1 and (**b**) Subject 2 between the reference system (Shimmer3 IMU, left column) and the prototype (Nicla Sense ME, right column). The three rows correspond to the X, Y, and Z accelerometer axes, respectively. The color scale represents the magnitude of the spectral components in m/s^2^, highlighting the temporal evolution of the dominant oscillation frequency during the five-phase protocol.

Figure 5 shows the amplitude spectrograms of the prototype (Nicla Sense ME) and the reference (Shimmer3 IMU) across the three accelerometer axes for Subject 1 and Subject 2. In both subjects, the spectrograms exhibit good agreement, showing step-like frequency variations consistent with the five-phase protocol.

For Subject 1 (Figure 5a), the spectrograms show a progressive increase in oscillation frequency across the phases: an initial low-frequency component around 1 Hz (Phase 1), followed by an increase to approximately 2 Hz (Phase 2), then to approximately 4 Hz (Phase 3), and finally to approximately 6 Hz in Phase 4. In the final relaxation phase (Phase 5), the oscillation frequency decreases and returns to approximately 2 Hz.

For Subject 2, the spectrograms appear visually clearer than those for Subject 1, consistent with a more regular and larger amplitude execution of the hand oscillations and, consequently, a higher signal-to-noise ratio. The phase progression is again evident: Phase 1 remains below 2 Hz, Phase 2 reaches approximately 2 Hz, Phase 3 is characterized by an oscillation around 5 Hz lasting about 50 s, Phase 4 reaches a maximum frequency of approximately 7 Hz and, in the final phase, the oscillation attenuates and returns to below 2 Hz.

Beyond qualitative inspection, the agreement between the prototype and the reference was quantified at two levels. First, the complete amplitude spectrograms were compared using the Pearson correlation coefficient, MAE, and RMSE. Second, the dominant-frequency trajectories extracted from the spectrograms were evaluated using correlation and error-based agreement metrics. The spectrogram metrics are reported in Table 3, while the dominant-frequency metrics are reported in Table 4.

Table 3 summarizes the comparison of the time–frequency representations over the band of interest. The Pearson correlation coefficients range from 0.68 to 0.85, showing a consistent correspondence between the spectral patterns measured by the prototype and the reference system. For Subject 1, the highest correlation is obtained on the Y axis (r=0.85), followed by the Z axis (r=0.82), while the X axis gives r=0.74. For Subject 2, the correlation ranges from 0.68 on the X axis to 0.79 on the Z axis.

The amplitude errors are generally limited. MAE ranges from 0.07 m/s^2^ to 0.19 m/s^2^, while RMSE ranges from 0.14 m/s^2^ to 0.66 m/s^2^. The smallest errors are observed for Subject 1 on the X axis (MAE = 0.07 m/s^2^, RMSE = 0.14 m/s^2^), whereas the largest values occur for Subject 2 on the Z axis (MAE = 0.19 m/s^2^, RMSE = 0.66 m/s^2^). The observed axis dependence may be related to differences in the relative motion components along the three sensing directions and to small residual misalignments between the axes of the two devices.

**Figure 6:**
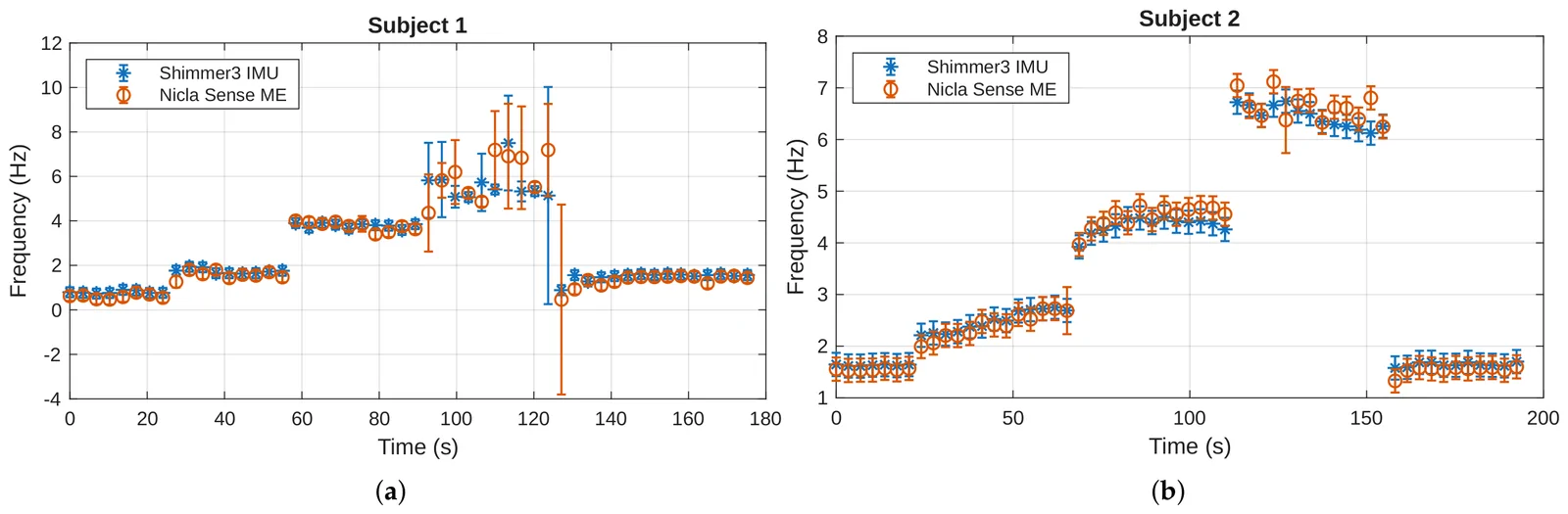
Dominant-frequency tracking comparison (Y axis) between the reference system (Shimmer3 IMU, blue markers) and the prototype (Nicla Sense ME, orange markers) for (**a**) Subject 1 and (**b**) Subject 2. The error bars represent the measurement uncertainty, estimated by propagating measurement repeatability and the sampling-time variability through a Monte Carlo approach.

A visual comparison of the estimated dominant-frequency vectors obtained with the Shimmer3 and the Nicla Sense ME is shown in Figure 6 for both subjects. The plots report, for each time window, the dominant-frequency component extracted from the spectrograms of the reference and prototype systems.

To improve the accuracy of the dominant-frequency estimation, a three-point interpolated DFT approach was applied to the acquired spectra. After identifying the maximum spectral sample within the frequency band of interest, the peak location was refined by quadratic interpolation using the peak bin and its two adjacent bins [28].

The plots in Figure 6 refer to the accelerometer data acquired along the Y axis. The error bars associated with each dominant-frequency estimate were evaluated by Monte Carlo propagation (10,000 trials) of the measurement repeatability and sampling-time variability. These two contributions were first estimated from dedicated time-domain acquisitions and then propagated to the frequency estimate. Since, for the Nicla Sense ME validation dataset, only spectral data were available, a full propagation through the complete measurement chain starting from the original time-domain signals was not possible. Therefore, an empirical spectral-dispersion model was first identified from ad hoc time-domain acquisitions carried out under the same operating conditions and processed through the same spectral chain used in the validation procedure. Specifically, the repeatability observed in the time-domain measurements, together with the timing variability estimated from the timestamps provided by the Nicla board, was propagated through the spectrogram computation in order to quantify the resulting variability of the spectra. The identified spectral-dispersion model was then used as the input quantity for a second Monte Carlo (10,000 trials) propagation performed directly on the measured spectra of the validation dataset, thereby obtaining the uncertainty contribution associated with the estimated dominant frequencies, which is represented in the plots with a coverage factor of k=2.

Table 4 reports the agreement between the dominant-frequency estimates obtained from the prototype and the reference system. The analysis includes the Pearson correlation coefficient, the signed mean error (bias), corresponding to the Bland–Altman bias, the standard deviation of the error (SD), MAE, RMSE, and the 95% limits of agreement (LoAs). The bias quantifies systematic frequency offsets, whereas MAE and RMSE quantify the magnitude of the estimation error. The LoA provide an estimate of the interval containing approximately 95% of the pairwise differences.

The results in Table 4 show a clear axis-dependent behavior. For Subject 1, the Y and Z axes exhibit strong agreement, with Pearson coefficients of 0.99 and 0.92, respectively. On these axes, the MAE values are 0.20 Hz and 0.34 Hz and the RMSE values are 0.26 Hz and 0.62 Hz. The corresponding Bland–Altman limits of agreement are also comparatively narrow, particularly for the Y axis ([−0.57,0.29] Hz). In contrast, the X axis shows lower correlation (r=0.59) and substantially larger dispersion, with an MAE of 1.52 Hz, an RMSE of 2.80 Hz, and 95% LoA of [−5.77,5.27] Hz.

For Subject 2, the agreement is more uniform. The Y and Z axes both reach a Pearson coefficient of 0.99, with identical MAE values of 0.17 Hz and RMSE values of 0.20 Hz. The X axis also shows a relatively high correlation (r=0.85), although the error dispersion is larger, with an MAE of 0.48 Hz and an RMSE of 1.18 Hz. Across all subject–axis combinations, the Bland–Altman biases remain between −0.252 and 0.048 Hz, indicating that the dominant-frequency discrepancies are mainly associated with dispersion rather than with a systematic over- or underestimation by the prototype.

To specifically evaluate the frequency-estimation performance within the typical Parkinsonian tremor band, Table 5 reports MAE and RMSE for the windows in which the dominant frequency measured by the Shimmer3 IMU fell within 4 Hz–7.5 Hz. The Y and Z axes show consistently small errors for both subjects: MAE ranges from 0.12 Hz to 0.15 Hz and RMSE from 0.15 Hz to 0.20 Hz. Larger errors are again observed on the X axis, with MAE/RMSE values of 1.47 Hz/2.53 Hz for Subject 1 and 0.59 Hz/1.40 Hz for Subject 2. It can be observed that the agreement is strongest on the axes carrying the most clearly defined oscillatory components, whereas the X axis is more sensitive to the relative distribution of the hand motion among the sensing directions.

## 4. Discussion

The results obtained from the comparative validation show that the proposed system is able to reproduce the main time–frequency characteristics of the hand oscillations measured by the Shimmer3 IMU. The spectrogram-based metrics show some variability across subjects and sensing axes; however, the absolute MAE and RMSE values should also be interpreted in relation to the amplitude of the measured acceleration signals. In particular, as shown in Figure 5, Subject 2 exhibited oscillations with generally larger acceleration amplitudes than Subject 1. Therefore, the apparently higher amplitude errors observed for some axes of Subject 2 do not necessarily indicate poorer relative performance of the prototype. The dominant-frequency results also show clear axis-dependent behavior, with the Y and Z axes generally providing the best agreement, whereas larger error dispersion is observed along the X axis. This can reasonably be related to the direction of the voluntary hand motion adopted during the validation protocol: as can be observed in Figure 5, the oscillatory components were more pronounced along the Y and Z axes, while the X axis contribution was substantially smaller. The lower signal amplitude along the X axis therefore resulted in a less clearly defined dominant spectral component and, consequently, in a less robust frequency estimation.

The prototype appears particularly effective in reproducing the location and temporal evolution of the dominant spectral components, as demonstrated by the high Pearson correlation coefficients and the low MAE and RMSE values obtained for dominant-frequency estimation, especially along the Y and Z axes. Conversely, the agreement in spectral amplitude is more limited. This behavior can reasonably be related, at least in part, to the present stage of development of the prototype. In particular, the accelerometer integrated into the Nicla Sense ME was not independently calibrated against a traceable acceleration reference within this study, and the conversion of the measured acceleration values relied on the factory calibration and sensitivity specifications provided by the sensor manufacturer. Consequently, residual scale-factor or offset errors may affect the absolute estimation of acceleration amplitude, while having a much smaller influence on the identification of the dominant-frequency component.

Beyond the direct comparison with the reference device, it is also useful to place the proposed system in the context of previously reported wearable solutions for tremor monitoring. Table 6 summarizes the main hardware and system-level characteristics of representative devices from the literature, allowing the proposed architecture to be compared in terms of sensing configuration, form factor, stand-alone operation, on-device spectral processing, dimensions, weight, battery life, and recording capability. The proposed prototype combines several features that are particularly relevant for continuous wearable monitoring. With overall dimensions of approximately 23×23×10 mm^3^ and a mass of only 6.3 g, it is among the most compact and lightweight solutions in the comparison for which these parameters are reported. At the same time, the device operates as a stand-alone measurement node, independently acquiring and storing the processed data without requiring a continuous connection to an external host. A further relevant feature is the on-board computation of the acceleration spectra: among the systems included in Table 6, the proposed prototype is the only one combining stand-alone operation with edge-based spectral processing. This allows the frequency-domain information relevant to tremor monitoring to be extracted directly on the wearable node, while BLE communication is required only when the stored data have to be transferred.

## 5. Conclusions

The aim of this study was to develop and validate a compact, low-cost wearable measurement system for continuous tremor monitoring through a metrological comparison with a commercial system. The system, based on the Arduino Nicla Sense ME board, was designed to acquire and store spectrograms computed in real time from signals provided by the integrated three-axis accelerometer.

The experimental validation of the proposed measurement system was conducted on two healthy subjects, who were asked to perform controlled voluntary hand oscillations at progressively varying frequencies according to a well-defined protocol. The explored frequencies ranged from values below 2 Hz to approximately 7 Hz, thus covering the frequency band associated with Parkinsonian tremor. However, the adopted protocol was intended primarily for technical comparison and was not designed to reproduce the full kinematic complexity of pathological tremor. The prototype was validated through comparison with a commercial reference device, the Shimmer3 IMU, which is widely used in clinical and human movement research applications. The comparison showed Pearson correlation coefficients of up to 0.99 for dominant-frequency estimation and 0.85 for the spectrogram comparison. For dominant-frequency estimation, MAE and RMSE values as low as 0.17 Hz and 0.20 Hz were obtained, respectively, while the Bland–Altman bias remained between −0.252 Hz and 0.048 Hz across all subject–axis combinations. These results indicate good agreement between the developed prototype and the commercial reference system despite the substantially lower cost of the proposed device.

Future developments will focus on exploiting the full IMU capabilities by integrating accelerometer, gyroscope, and magnetometer data. This may enable the investigation of additional movement-related parameters beyond tremor frequency through a dedicated validation against a reference system. The system will then be tested on a larger cohort, including subjects with neurological disorders, to assess its performance under clinically relevant conditions. Moreover, the system will be tested at different body locations and under different postural conditions, since these factors may affect the relative contribution of motion along the different sensing axes, the mechanical coupling between the sensor and the body, and the susceptibility to motion artifacts, as also reported in previous studies [33]. In addition, further work will address device scalability, with the possibility of deploying multiple wearable nodes on the same patient to enable synchronous multi-site data acquisition. Finally, the device will be tested on a larger cohort of subjects and under medical supervision to further assess its clinical applicability and robustness.

## Figures and Tables

**Table 1:** Technical specification for the Bosch BHI260AP [25].

Parameter	Typical Value
Acceleration Range	±2 g, ±4 g, ±8 g, ±16 g
Resolution	16 bit
Sensitivity (FS = 4 g)	8192 LSB/g
Sensitivity Error	±0.8%
Zero-g Offset	±50 mg
Nonlinearity	±0.5% FS mg
Output noise	1.2 mg-rms
Cross Axis Sensitivity	1%

**Table 2:** Comparison between the commercial Shimmer3 IMU [26] and the developed prototype.

Parameter	Shimmer3 IMU	Developed Prototype
Dimensions	51 × 34 × 14 mm^3^	23 × 23 × 10 mm^3^
Weight	23.6 g	6.3 g
Battery capacity	450 mAh	150 mAh
Battery lifetime	14 h	20 h
Range	±2 g, ±4 g, ±8 g, ±16 g	±2 g, ±4 g, ±8 g, ±16 g
Resolution	16 bit	16 bit
Cost	430 euro	<100 euro

**Table 3:** Spectrogram similarity metrics between prototype and reference, reported by subject and axis.

Subject	Axis	Pearson *r*	MAE (m/s^2^)	RMSE (m/s^2^)
Subject 1	X	0.74	0.07	0.14
	Y	0.85	0.10	0.24
	Z	0.82	0.08	0.19
Subject 2	X	0.68	0.10	0.30
	Y	0.78	0.16	0.39
	Z	0.79	0.19	0.66

**Table 4:** Dominant-frequency comparison between the prototype and the reference system. The mean error corresponds to the Bland–Altman bias, while the 95% limits of agreement (LoAs) are calculated as bias±1.96SD.

Subject	Axis	Pearson *r*	Bias (Hz)	SD (Hz)	MAE (Hz)	RMSE (Hz)	95% LoA (Hz)
Subject 1	X	0.59	−0.252	2.81	1.52	2.80	[−5.77,5.27]
	Y	0.99	−0.139	0.22	0.20	0.26	[−0.57,0.29]
	Z	0.92	−0.119	0.62	0.34	0.62	[−1.33,1.10]
Subject 2	X	0.85	0.048	1.19	0.48	1.18	[−2.29,2.38]
	Y	0.99	−0.052	0.20	0.17	0.20	[−0.44,0.33]
	Z	0.99	−0.039	0.19	0.17	0.20	[−0.42,0.34]

**Table 5:** Dominant-frequency estimation errors within the typical Parkinsonian tremor band (4 Hz–7.5 Hz).

Subject	Axis	MAE (Hz)	RMSE (Hz)
Subject 1	X	1.47	2.53
	Y	0.12	0.15
	Z	0.14	0.18
Subject 2	X	0.59	1.40
	Y	0.15	0.20
	Z	0.14	0.18

**Table 6:** Comparative summary of tremor-assessment systems. In the “Sensors” column, A denotes an accelerometer, G a gyroscope, and M a magnetometer. N/A indicates that the corresponding information was not reported in the cited study.

Ref	Year	Form Factor	Sensors	Placement	Sampling Rate (Hz)	Edge Spectral Proc.	Stand-Alone	Dimensions	Weight	Battery Life	Max. Recording Duration
[29]	2015	Glove	A + G	Finger	100	No	No	N/A	N/A	N/A	Host-dependent
[30]	2017	Wearable IMU	A + G + M	Waist	50	No	Yes	99×53×19 mm^3^	83 g	∼91 h	∼91 h
[31]	2017	Smartwatch	A	Wrist	25	No	No	N/A	N/A	N/A	N/A
[19]	2019	Wearable device	A	Fingers	50	No	Yes	60×110×30 mm^3^	N/A	∼48 h	SD-dependent
[32]	2020	Wrist-worn device	A	Wrist	100	No	No	28×18 mm^2^ (PCB)	36 g	∼20 h	N/A
[20]	2020	Wearable device	A + G + M	Hand dorsum	50	No	No	25×25 mm^2^	N/A	N/A	N/A
[27]	2021	Smartwatch	A	Wrist	50	No	Yes	46.6×51.8×12.9 mm^3^	32.5 g	N/A	>100 h
[17]	2025	Wearable device	A + G	Finger	55.56	No	Yes	N/A	N/A	N/A	microSD-dependent
[12]	2025	Smartwatch	A + G	Wrist	100	No	No	38.6×33.3×11.4 mm^3^	N/A	N/A	N/A
This work	2026	Wearable device	A	Hand dorsum	50	Yes	Yes	23×23×10 mm^3^	6.3 g	∼20 h	∼5 h

## Data Availability

Data are contained within the article.
